# Incomplete Left Atrial Appendage Closure: How to Address the (Knowledge) Gap?

**DOI:** 10.1016/j.shj.2025.100466

**Published:** 2025-06-24

**Authors:** Yannick Willemen, Ole De Backer

**Affiliations:** The Heart Centre, Rigshospitalet, Copenhagen University Hospital, Copenhagen, Denmark

Left atrial appendage (LAA) closure is an established strategy for stroke prevention in selected patients with atrial fibrillation (AF).^1^^,^^2^ Following the American and European guidelines, transcatheter LAA closure may be considered for AF patients who are at moderate to high risk of thromboembolism but with contraindications to long-term oral anticoagulation (OAC).^1^, ^2^, ^3^ In addition, surgical LAA ligation or excision is recommended as an adjunct to OAC in AF patients undergoing cardiac surgery.^1^^,^^2^ Regardless of the approach, complete LAA closure should always be the goal, as incomplete transcatheter or surgical LAA closure has been associated with increased risk of thromboembolism.^4^^,^^5^ Unfortunately, incomplete LAA closure, including peri-device leak (PDL) and/or an LAA remnant, is not an infrequent finding during imaging follow-up. However, how to assess it, its clinical significance, and optimal management strategies remain largely unclear and a matter of debate.^3^^,^^6^

In this issue of *Structural Heart*, Richardson et al.^7^ report on the completeness of surgical LAA exclusion using different techniques in a single-center patient cohort treated between 2012 and 2019, based on retrospective evaluation of transesophageal echocardiography (TEE) imaging performed during postoperative follow-up. Successful LAA exclusion, defined as an LAA remnant maximum depth <1 cm, was obtained in 82% of all patients. Only 69% of the patients was free of any potential marker of suboptimal LAA exclusion on TEE, which included color-Doppler flow into the LAA remnant, residual pectinate muscle, visible suture, or thrombus. There was no statistically significant difference in the rates of successful LAA exclusion when comparing different surgical techniques that were used. In summary, nearly one-fifth of patients who underwent surgical LAA closure had an LAA remnant depth ≥1 cm, while nearly one-third had at least one TEE characteristic of suboptimal LAA closure. These findings reiterate that incomplete LAA closure is common after surgical LAA closure.

Notwithstanding the unique contribution of this study to a better understanding of this field, this study has several limitations that should be considered when interpreting its findings. First, this was a relatively small (N = 121), single-center study using different surgical techniques (surgical excision, AtriClip occlusion, or Tiger Paw occlusion). Second, TEE imaging during follow-up was not routine but rather performed in selected patients as part of OAC decision-making; this may have introduced some patient selection bias. Third, if relevant TEE image views were not recorded at the time, they could not be reviewed retrospectively, limiting the assessment of any LAA remnant. This would have been different in case contrast-enhanced cardiac computed tomography (CT) would have been available. Another limitation is the lack of data on OAC treatment. Imaging follow-up was reportedly commonplace when considering OAC strategy, yet the study does not provide information on how OAC was managed postoperative, especially in relation to imaging findings. The OAC strategy could be particularly relevant given that it can be advocated to initiate OAC in case of a significant PDL or LAA remnant. Without this information on OAC use, the reported stroke rates after successful versus unsuccessful LAA exclusion are impossible to interpret and merely descriptive.

In the surgical field, the Left Atrial Appendage Occlusion Study III trial provided landmark evidence supporting surgical LAA occlusion for stroke prevention in AF patients undergoing cardiac surgery.^8^ However, many questions remain, especially regarding the optimal patient selection, the long-term durability of different surgical techniques, and the impact of incomplete LAA exclusion. A parallel can be drawn to the transcatheter LAA closure field, where the same uncertainties exist.^4^^,^^6^ The importance of complete LAA closure has been increasingly recognized, as residual flow into the LAA has been associated with increased thromboembolic risk, thereby mitigating the anticipated stroke prevention benefit of LAA closure.

The study findings by Richardson et al.^7^ reiterate and emphasize that one of the key points and challenges in both surgical and transcatheter LAA closure is accurate assessment of the LAA closure result. While TEE remains the standard for periprocedural evaluation, there has been an evolution toward using three-dimensional imaging because it provides more precise LAA characterization. Both three-dimensional TEE and cardiac CT are increasingly used for preprocedural planning and postprocedural follow-up of transcatheter LAA closure. Studies have demonstrated that preprocedural CT imaging increases the likelihood of implantation success, and postprocedural CT imaging enhances the detection of PDL compared to TEE, particularly for small leaks that may be missed on TEE imaging.^9^^,^^10^ However, also the clinical significance of small residual leak or LAA patency remains debated. In a large meta-analysis, any PDL detected by TEE after LAA closure was associated with a 1.8-fold increase in thromboembolic risk, and PDL ≥3-5 mm was associated with a 4-fold higher risk.^4^ On the other hand, LAA patency detected by CT (ie, contrast visible in the LAA distal of an implanted device) was not significantly associated with an increased thromboembolic risk.

Finally, the presence of a significant PDL or LAA remnant raises concerns regarding thromboembolic risk, but management strategies remain debated. Potential approaches include extended OAC or tailored antithrombotic regimens for patients with persistent leaks, although balancing the bleeding and thromboembolic risk may be challenging.^3^^,^^6^ Other case series and studies investigated secondary occlusion techniques, including plugs, coils, or additional closure devices.^3^^,^^6^ Clinical experience with these methods is growing, but data on long-term outcomes are still missing and needed.^6^ Surgical reintervention may be considered for select patients undergoing repeat cardiac surgery; however, this will only be considered in rare cases with a truly suboptimal LAA exclusion result. Overall, the threshold for reintervention remains high and unclear. The lack of consensus in this matter underscores the need for further research into the mechanism of leaks and the associated thromboembolic risk, to define risk-based strategies tailored to individual patient profiles.

Moving forward, standardized cardiac imaging protocols (from preprocedural planning to routine postprocedural follow-up), long-term outcome data, and randomized controlled trials comparing different LAA closure techniques and incomplete LAA closure management strategies will be crucial. The field of transcatheter LAA closure is expanding rapidly, with newer devices and procedural refinements aiming to improve procedural safety and effectiveness. At the same time, surgical LAA occlusion must also continue to be improved, ensuring that patients—also without history of AF—undergoing cardiac surgery receive the best possible stroke prevention strategy.

In conclusion, the study by Richardson et al contributes to our knowledge about surgical LAA closure, but it also reiterates critical questions about optimal imaging follow-up strategies and the clinical significance and management of LAA remnants or leaks. As both surgical and transcatheter LAA closure continue to advance as important strategies for stroke prevention in AF patients, the focus should remain on achieving complete and durable LAA closure. Future research should aim to harmonize preprocedural, periprocedural, and postprocedural imaging protocols, and investigate and refine residual LAA remnant or leak management strategies in order to optimize clinical outcomes.

## Funding

The authors have no funding to report.

## Disclosure Statement

O. De Backer received institutional research grants and consulting fees from Abbott, Boston Scientific, and Medtronic. The other author had no conflicts to declare.
